# Supplementary material for: A Novel, Circulating Tumor Cell Enrichment Method Reduces ARv7 False Positivity in Patients with Castration-Resistant Prostate Cancer
Source: Diagnostics (Basel). 2020 Mar 11;10(3):151. doi: 10.3390/diagnostics10030151 (PMC7151149; doi:10.3390/diagnostics10030151)
Supplement: Supplementary file 1 [file diagnostics-10-00151-s001.pdf]

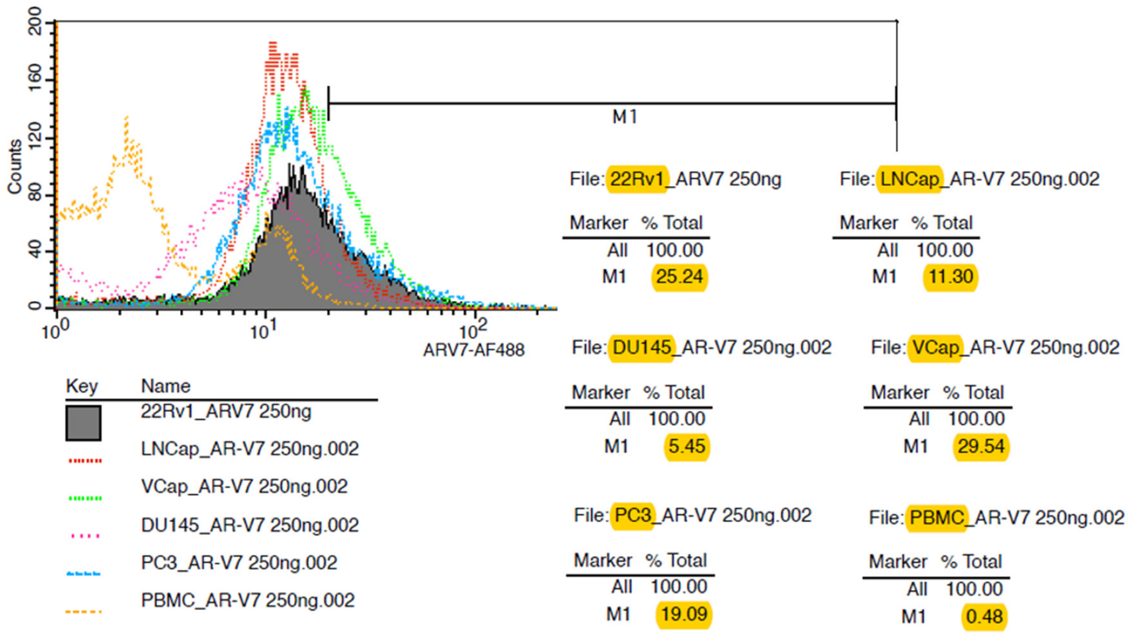


**Figure 1.** Evaluation of AR-V7 antibody reactivity in fluorescence-activated cell sorting (FACS).

We verified that the antibody response of the anti-AR-V7 antibody (EPR15656; Abcam) conjugated AF-488 in prostate cancer cell lines, using FACS (BD FACSCalibur HG, BD Biosciences). We confirmed positive responses of 29.54% and 25.24% in Vcap (ATCC CRL-2876) and 22RV1 (ATCC CRL-2505) cell lines, respectively; these are normally positive for AR-V7. However, the positivity for AR-V7-negative cell lines DU145 (ATCC HTB-81), PC3 (ATCC CRL-1435), and LNCap (ATCC CRL-1740) had positive responses of 5.45%, 19.09%, and 11.30%, respectively. Moreover, PBMCs showed a positive response of 0.48%.
